# E-selectin-targeting delivery of microRNAs by microparticles ameliorates endothelial inflammation and atherosclerosis

**DOI:** 10.1038/srep22910

**Published:** 2016-03-09

**Authors:** Shuangtao Ma, Xiao Yu Tian, Yunrong Zhang, Chaofeng Mu, Haifa Shen, Jean Bismuth, Henry J. Pownall, Yu Huang, Wing Tak Wong

**Affiliations:** 1 Department of Cardiovascular Sciences, Houston Methodist Research Institute, Houston, TX 77030, USA; 2 Institute of Vascular Medicine, School of Biomedical Sciences and Li Ka Shing Institute of Health Sciences, Chinese University of Hong Kong, Shatin, NT, Hong Kong SAR, China; 3 Department of Nanomedicine, Houston Methodist Research Institute, Houston, TX 77030, USA; 4 Methodist DeBakey Heart & Vascular Center, Houston Methodist Hospital, Houston, TX 77030, USA

## Abstract

E-selectin is a surface marker of endothelial cell (EC) inflammation, one of the hallmarks of atherogenesis. Thus, we tested the hypothesis that delivery of microRNA (miR)-146a and miR-181b with an E-selectin-targeting multistage vector (ESTA-MSV) to inflamed endothelium covering atherosclerotic plaques inhibits atherosclerosis. Cy5-conjugated miR-146a and miR-181b were packaged in polyethylene glycol-polyethyleneimine (PEG/PEI) nanoparticles and loaded into ESTA-MSV microparticles. Both miRs were downregulated in tumor necrosis factor (TNF)-α-treated ECs. Transfection of TNF-α-treated mouse aortas and cultured ECs with miRs was more efficient with ESTA-MSV than with the PEG/PEI. Likewise, miR-146a/-181b packaged in ESTA-MSV efficiently suppressed the chemokines, CCL2, CCL5, CCL8, and CXCL9, and monocyte adhesion to ECs. Complementary *in vivo* tests were conducted in male apolipoprotein E-deficient mice fed a Western diet and injected intravenously with the particles prepared as above biweekly for 12 weeks. Treatment with miRs packaged in ESTA-MSV but not in PEG/PEI reduced atherosclerotic plaque size. Concurrently, vascular inflammation markers, including macrophages in aortic root lesions and chemokine expression in aortic tissues were reduced while the vascular smooth muscle cells and collagen increased in plaques from ESTA-MSV/miRs-treated *vs.* vehicle-treated mice. Our data supported our hypothesis that ESTA-MSV microparticle-mediated delivery of miR-146a/-181b ameliorates endothelial inflammation and atherosclerosis.

Atherosclerosis is a chronic inflammatory disease of the arterial wall. Endothelial dysfunction and inflammation are essential to the initiation and progression of atherosclerosis^1^. Atherosclerotic lesions are more abundant and severe in the regions of low shear stress, an important risk factor for atherosclerosis^2^. New mediators including cytokines, surface molecules, enzymes, transcription factor, and post-transcriptional mediators have been incorporated into refined mechanistic models of low shear stress-induced endothelial inflammation. The microRNAs (miRs) are small non-coding RNAs involved in post-transcriptional gene regulation^3^; miRs mediate the effects of shear stress on the endothelium^4^,^5^. Several high shear stress-inducible miRs, such as miR-10a, miR-19a, and miR-23b, have been identified as atheroprotective^6^,^7^,^8^. Moreover, exposure of cultured endothelial cells (ECs) to laminar shear stress upregulated miR-146a and miR-181b suggesting that both miRs have potential atheroprotective effects^6^,^9^. MiR-146a and miR-181b respectively also exert anti-inflammatory effects by directly targeting to the 3′-untranslated region (3′-UTR) of tumor necrosis factor (TNF) receptor-associated factor 6 (TRAF6) and importin α3, subsequently inhibiting nuclear factor-kappa B (NF-κB) activity. This is highly relevant given that NF-κB is a regulator of chemokines expression. These include CCL2, CCL5, CCL8, and CXCL9, which mediate the adhesion of monocytes to ECs. Given the fundamental role of monocyte adhesion in atherogenesis^10^, their targeting with miRs could suppress endothelial inflammation and atherosclerosis.

Efficient tissue-specific delivery of therapeutic miRs *in vivo* is a major challenge for miR-based therapy^11^. Nanoparticles have emerged as a promising vector for miR delivery^12^, and polyethylene glycol-polyethyleneimine (PEG/PEI) nanoparticles have been validated as an efficient delivery vector^13^. However, rapid clearance and poor targeting limit their clinical value. We developed and tested a porous silicon multistage vector (MSV) delivery system, which is a micrometer-sized nanoporous microparticle^14^. Large amounts of therapeutic agents have been packaged into PEG/PEI nanoparticles and loaded into the nanopores within the MSV microparticles. Once reaching its target, the slow degradation of the silicon carrier into a non-toxic orthosilicic acid coincides with the sustained release of the therapeutic agent. Adhesion molecules, such as E-selectin, not only facilitate the monocyte adherence to ECs but are also attractive docking sites for inflamed endothelium-targeted drug delivery. We developed a thioaptamer (ESTA) that binds specifically to E-selectin and covalently conjugated the ESTA onto the surface of a MSV to form an ESTA-MSV targeting delivery system, which efficiently delivers siRNA to tumor-inflamed endothelium^15^. Thus, we hypothesized that the ESTA-MSV microparticle specifically and efficiently deliver miR-146a/-181b to inflamed endothelium on the luminal face of atherosclerotic lesions. The present study tested the feasibility of delivering miR-146a/-181b by affinity targeting to E-selectin and its effectiveness for the reduction of endothelial inflammation and atherosclerosis in apolipoprotein E-deficient (ApoE^−/−^) mice.

## Methods

### Reagents

The mature miR-146a (5′-UGA GAA CUG AAU UCC AUG GGU U-3′), miR-181b (5′-AAC AUU CAU UGC UGU CGG UGG GU-3′), and Cy5-labeled miRs were synthesized by Integrated DNA Technologies (IDT, Coralville, IA).

### Packaging miRs into nanoparticles or microparticles

Discoidal MSV microparticles were fabricated by electrochemical etching of silicon wafer and surface modified with 3-aminopropyltriethoxysilane (APTES) as previously described^16^. E-selectin thioaptamer was chemically conjugated to the APTES using 1-ethyl-3-(3-dimethylaminopropyl) carbodiimide hydrochloride as a polylinker^15^. The PEG/PEI/miRs nanoparticles and ESTA-MSV/miRs microparticles were prepared as previously described^15^. In brief, the polyplexes were prepared with 15:1 ratio between nitrogen in cationic polymer and phosphorus in miR oligo (N/P ratio). MiRs were first mixed with PEG(5k)2–PEI(10k) (PEG–PEI), and then incubated at 20 °C for 15 min to form the polyplexes. The PEG/PEI/miRs polyplexes were then loaded into the ESTA-MSV particles by sonication in a water bath for 3 min. The ESTA-MSV particles were fabricated as described^15^. ESTA-MSV particles were then sedimented at 12,000 rpm for 5 min, and the supernatant containing excess PEG/PEI polyplexes removed.

### Cell culture

Human microvascular endothelial cells (HMVECs) were obtained from Lonza (Walkersville, MD) and cultured in EC growth medium EGM-2 MV (cc-3162). Cells were used for all experiments at passage 6–8. Cells were exposed to recombinant human TNF-α (10 ng/mL, R&D Systems, Abingdon, UK) for 2 or 6 h. The cells were transfected by a 24-h incubation with particles loaded with miR mimetics at 20 nmol/L, and then harvested for experiments.

### Cell adhesion assay

Transfected HMVECs were plated into 96-well plate overnight and then treated with TNF-α for 6 h. The human monocytic cell line, THP-1, was obtained from American Type Culture Collection (ATCC) and cultured in ATCC-formulated RPMI 1640 medium (GIBCO, Gaithersburg, MD) containing 10% fetal bovine serum. THP-1 cells were labeled with CellTracker Green CMFDA Dye (Life Technologies, Gaithersburg, MD) and washed twice with RPMI 1640 medium, and then 1 × 10^5^ labeled cells were added to the EC monolayer, and incubated in a CO_2_ incubator for 1 h on a rocker. Nonadherent cells were removed from the plate by gentle washing with PBS, and the number of THP-1 cells per view was also quantified from randomly acquired images.

### *Ex vivo* aorta transfection

C57BL/6J (Jackson Laboratory) mice were sacrificed by CO_2_ anesthesia. The aortas were freed from the surrounding connective tissue using a dissecting stereomicroscope. The vessels were cut into small segments (~2 mm length) and opened longitudinally with fine scissors and placed in 96-well plate. The *en face* preparations of aortas were cultured in Dulbecco’s Modified Eagle Medium (DMEM, GIBCO) and transfected with Cy5-labeled miRs packaged in particles as described above for 24 h. The mean fluorescence intensity of Cy5 (Ex/Em = 650 nm/670 nm) was measured using an Infinite M1000 microplate reader (Tecan, Mechelen, Belgium).

### *In vivo* delivery of miRs in mice

All protocols concerning animal use were approved by the Institutional Animal Care and Use Committee (IACUC) of Houston Methodist Research Institute and the methods were carried out in accordance with the approved guidelines. The 8-week-old male ApoE^−/−^ mice, purchased from Jackson Laboratory, were fed a Western diet (TD.88137; Harlan Laboratories Inc., Indianapolis, IN) for 12 weeks. Meanwhile, mice were injected with PEG/PEI-vehicle, PEG/PEI-miR-146a, PEG/PEI-miR-181b nanoparticles, or ESTA-MSV-vehicle, ESTA-MSV-miR-146a, ESTA-MSV-181b microparticles biweekly for 12 weeks via the tail vein. Each particle injection contained 15 μg miRs in 100 μL, per mouse.

### Intraperitoneal glucose tolerance test (IPGTT)

After 12-week feeding and particle injection, the IPGTT was performed as described^17^. Briefly, mice were fasted overnight for 12 h and then injected intraperitoneally with glucose (1 g/kg body weight). The tail vein blood glucose level was measured with an automated glucometer (Bayer, Elkhart, IN) at baseline and 15, 30, 60, and 120 min after the injection.

### Analysis of serum parameters

Serum triglyceride (2200–430), total cholesterol (1010–430), low-density lipoprotein (LDL) cholesterol (0710–080), and high-density lipoprotein (HDL) cholesterol (0599–020) were measured using commercially available kits (Stanbio Laboratory, Boerne, TX) according to the manufacturer’s instruction. Serum alanine aminotransferase (ALT) activity, aspartate aminotransferase (AST) activity, and creatinine were measured using kits (MAK052, MAK055, MAK080, Sigma-Aldrich, St. Louis, MO).

### Vascular reactivity assays

Carotid arteries and abdominal aortas were dissected and cut into 2-mm-long segments in cold Krebs solution containing (in mmol/L): NaCl 119; NaHCO_3_ 25; KCl 4.7; KH_2_PO_4_ 1.2; MgSO_4_ 1.2; CaCl_2_ 2.5, and glucose 11.1; pH 7.4^18^. The artery rings were mounted in wire myograph chambers (620 M, DMT, Aarhus, Denmark) containing 5 mL of Krebs solution gassed with 95% O_2_ and 5% CO_2_ at 37 °C. The resting tension was adjusted to 3 mN for aortas and 1 mN for carotid arteries, and the bath solution replaced every 15 min. After a 60-min equilibration period, the viability of each ring was tested by exposure to a high-K^+^ Krebs solution (60 mmol/L KCl), and rings were allowed to rest in the organ bath for 30 min before any drugs were administered. To measure the relaxation response, a sustained contraction was induced by adding 3 μmol/L phenylephrine (Phe). Relaxations caused by incremental doses of acetylcholine (ACh, 10^−9^–10^−4^ mol/L) and sodium nitroprusside (SNP, 10^−9^–10^−4^ mol/L) were recorded. For measuring the endothelium-dependent contraction, carotid arteries were incubated with N-nitro-L-arginine methyl ester (L-NAME, 100 μmol/L, Sigma-Aldrich) for 30 min. Then, incremental doses of ACh (10^−9^–10^−4^ mol/L) were added to induce the contraction. Vasorelaxant responses are expressed as percent reduction in tone induced by Phe. Vasoconstriction responses are expressed as percent contraction in tone induced by 60 mmol/L KCl.

### *En face* staining of aorta

Mice were sacrificed by excess inhalation of carbon dioxide. Hearts and aortas were perfused *in situ* with phosphate-buffered saline (PBS) and then removed and placed on ice cold PBS. The perivascular fat and connective tissue around the aorta were carefully cleaned, and the aortic arch and thoracic aorta were cut longitudinally, fixed with isopropanol, and stained with oil red O (ORO) for 15 min. The images were captured by a digital camera.

### Histology

Mouse hearts with the aortic roots, livers, kidneys, and human aortic tissues were embedded in Tissue-Tek optimal cutting temperature (O.C.T.) medium (Sakura Finetek, Torrance, CA). Serial sections were cut at 6 μm thickness using a cryostat (CM1850, Leica, Nussloch, Germany). The mouse aortic root sections were stained with ORO and Masson’s trichrome (ab150686, Abcam, Cambridge, MA). The macrophages and vascular smooth muscle cells (SMCs) within the mouse aortic root lesions were identified by immunofluorescence assays. Frozen sections were fixed in acetone for 10 min, blocked in 5% normal donkey serum in PBS for 60 min, incubated at 4 °C with anti-CD68 antibody (1:100 dilution, MCA1957, AbD Serotec, Raleigh, NC) and anti-α-smooth muscle (SM)-actin antibody (1:100 dilution, A2547, Sigma-Aldrich). CD68 and α-SM-actin were visualized with Alexa Fluor 488 donkey anti-rat IgG secondary antibody (1:500 dilution, A21208, Life Technologies, Gaithersburg, MD) and Alexa Fluor 546 donkey anti-mouse IgG secondary antibody (1:500 dilution, A10036, Life Technologies), respectively. Cell nuclei were stained with Hoechst 33342 (Sigma-Aldrich). Images were acquired on a confocal microscope (FV1000-IX81, Olympus, Tokyo, Japan). The human aortic sections were stained with anti-platelet endothelial cell adhesion molecule 1 (PECAM1) antibody (1:50 dilution, BBA18, R&D Systems, Abingdon, UK) and anti-E-selectin antibody (1:100 dilution, 9G11, R&D Systems), and visualized with Alexa Fluor 488 donkey anti-mouse IgG secondary antibody (1:500 dilution, A21202, Life Technologies) and Alexa Fluor 594 donkey anti-goat IgG secondary antibody (1:500 dilution, A11058, Life Technologies), respectively. The frozen sections of mouse liver and kidney were stained with hematoxylin and eosin with a kit (9990001, Fisher Scientific, Pittsburgh, PA).

### Real-time qPCR

Total RNA was extracted using TRIzol reagent (Invitrogen, Carlsbad, CA) from homogenized mouse abdominal aortic tissues, human aortic tissues, or cells. The Quantitect reverse transcription kit (Qiagen, Chatsworth, CA, USA) was used to generate cDNA and SYBR Green PCR kit (Invitrogen) was used for real-time qPCR with the QuantStudio 12k Flex system (Applied Biosystems, Foster City, CA) following the manufacturer’s instructions. Primers are listed as follow: human E-selecin, vascular cell adhesion molecule 1 (VCAM1), intercellular adhesion molecule 1 (ICAM1), CCL2, CCL5, CCL8, CXCL9, and GAPDH, and mouse Ccl2, Ccl5, Ccl8, Cxcl9, and Gapdh are listed in Supplementary Table 1. To amplify mature miRNA sequences, TaqMan MicroRNA Assays kits including hsa-miR-146a (000468), hsa-miR-181b (001098), U6 snRNA (001973), TaqMan MicroRNA Reverse Transcription kit (P/N 4366596), and TaqMan Universal PCR Master Mix (P/N 4304437) were used.

### Statistical analysis

Data are presented as means ± SEM. Comparisons between groups were determined by one-way ANOVA with Student’s t-test post hoc test (SPSS Inc., Chicago, IL, USA). Results were considered significant when *P* value was less than 0.05.

## Results

### MiR-146a and miR-181b are downregulated in inflamed endothelium

**Figure 1:**
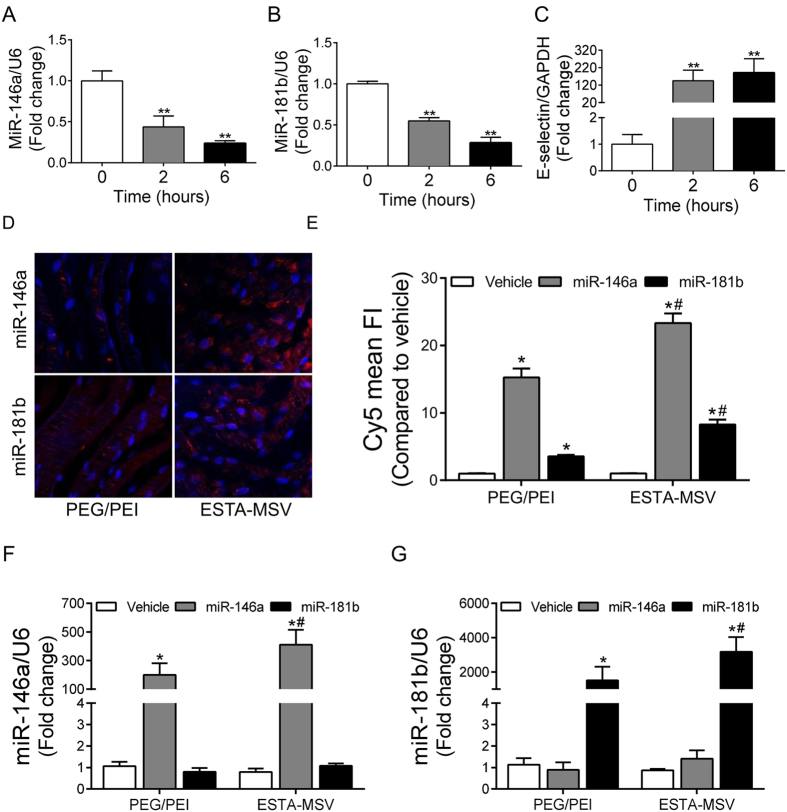
The expression of miR-146a and miR-181b in inflamed endothelial cells and the transfection efficiency of particles. The expression of miR-146a (**A**), miR-181b (**B**), and E-selectin (**C**) in HMVECs after treatment with TNF-α (10 ng/mL) for 0, 2, and 6 hours. **P < 0.01 vs. 0h. (**D**) Representative fluorescence images of TNF-α-treated en face mouse aortas transfected with PEG/PEI nanoparticles or ESTA-MSV microparticles containing Cy5-labeled miR-146a and miR-181b. Magnification: 40X. (**E**) Quantification of Cy5 mean fluorescence intensity. The expression of miR-146a (**F**) and miR-181b (**G**) in HMVECs after transfected with the particles as above. Data are shown as the means ± SEM and are representative of 3 independent experiments. *P < 0.05 vs. vehicle groups of the same particle; ^#^*P* < 0.05 vs. PEG/PEI groups of the same miR.

Induction of inflammation in HMVECs with TNF-α (10 ng/mL) significantly reduced miR-146a and miR-181b expression (Fig. 1A,B); miR-146a and miR-181b were also down regulated in human aortic tissue with plaque compared to the tissue without plaque (Supplementary Fig. S1A,B). We confirmed the expression of E-selectin (Fig. 1C), VCAM1, (Supplementary Fig. S2A) and ICAM1 (Supplementary Fig. S2B) was increased in inflamed HMVECs. Moreover, according to immunofluorescence of human aortic sections, E-selectin expression on irregular, inflamed endothelium covering plaques was enhanced compared to plaque-free endothelium (Supplementary Fig. S3).

### ESTA-MSV enhances transfection efficiency

We transfected the TNF-α-treated C57BL/6J mouse aortas with PEG/PEI nanoparticles and ESTA-MSV microparticles loaded with Cy5-conjugated miRs, and according to the mean fluorescence intensity, transfection efficiency was significantly higher in microparticle vs. nanoparticle groups (Fig. 1D,E). Transfection of the TNF-α-treated HMVECs with same particles revealed that both markedly increased the miR levels. Importantly, the miR levels were higher in microparticle- vs. nanoparticle-transfected ECs (Fig. 1F,G).

### ESTA-MSV/miRs inhibit adhesion molecule expression and function

**Figure 2:**
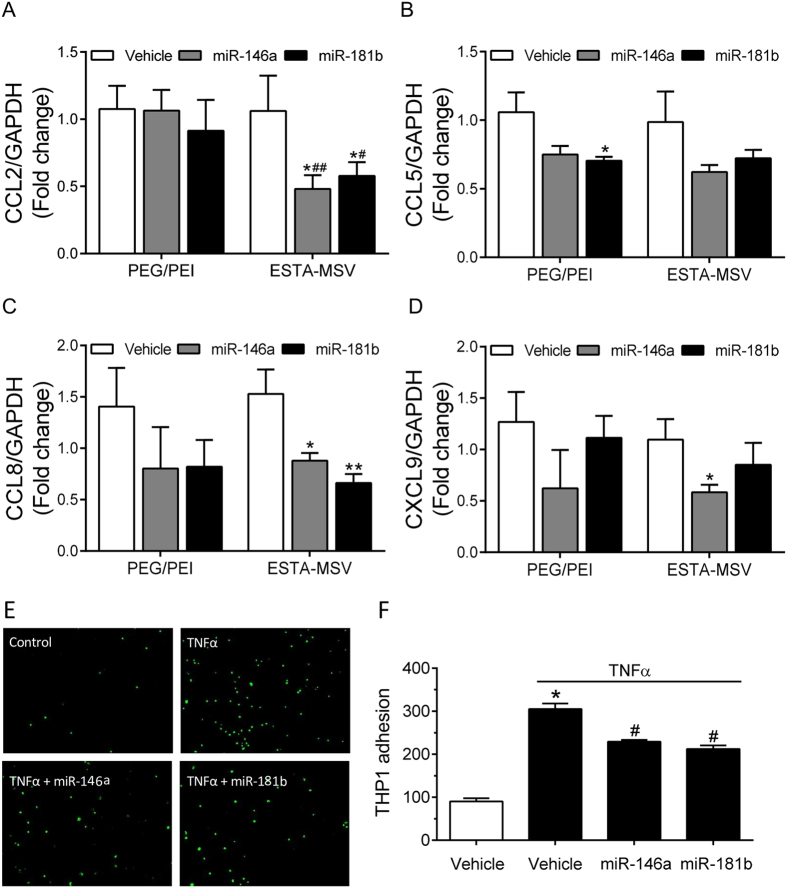
Effects of PEG/PEI/miRs and ESTA-MSV/miRs on the expression and function of adhesion molecules. The expression of CCL2 (**A**), CCL5 (**B**), CCL8 (**C**), and CXCL9 (**D**) in HMVECs after treated with TNF-α (10 ng/mL) and transfected with vehicle or miR-146a/-181b (20 nmol/L) loaded PEG/PEI nanoparticles and ESTA-MSV microparticles for 24 hours. *P < 0.05, **P < 0.01 *vs.* vehicle groups of the same particle. ^#^*P* < 0.05, ^##^*P* < 0.01 *vs.* PEG/PEI groups of the miR. (**E**) Representative images of Cell Tracker Green labeled THP1 monocytes adhered to monolayer of HMVECs treated with TNF-α and transfected with the particles as above for 24 hours. Magnification: 10X. (**F**) Quantified cell numbers of adhered THP1 cells. Data are shown as the means ± SEM and are representative of 3 independent experiments. *P < 0.05 vs. vehicle group without TNF-α, ^#^*P* < 0.05 vs. vehicle group with TNF-α.

Chemokines including CCL2, CCL5, CCL8, and CXCL9 mediate the adhesion of monocytes to ECs. Microparticle transfection of HMVECs for 24 h significantly reduced CCL2, CCL8, and CXCL9 expression (Fig. 2A–D). In contrast, PEG-PEI/miRs inhibited the expression of CCL5, but not the major chemokine CCL2 (Fig. 2B). Consistent with these data, ESTA-MSV/miRs also inhibited the TNF-α-induced adhesion of monocytes to ECs (Fig. 2E,F), indicating that microparticles delivers miRs more efficiently than PEG/PEI nanoparticles alone *in vitro*.

### ESTA-MSV/miRs improve endothelial function of ApoE^−/−^ mice

**Figure 3:**
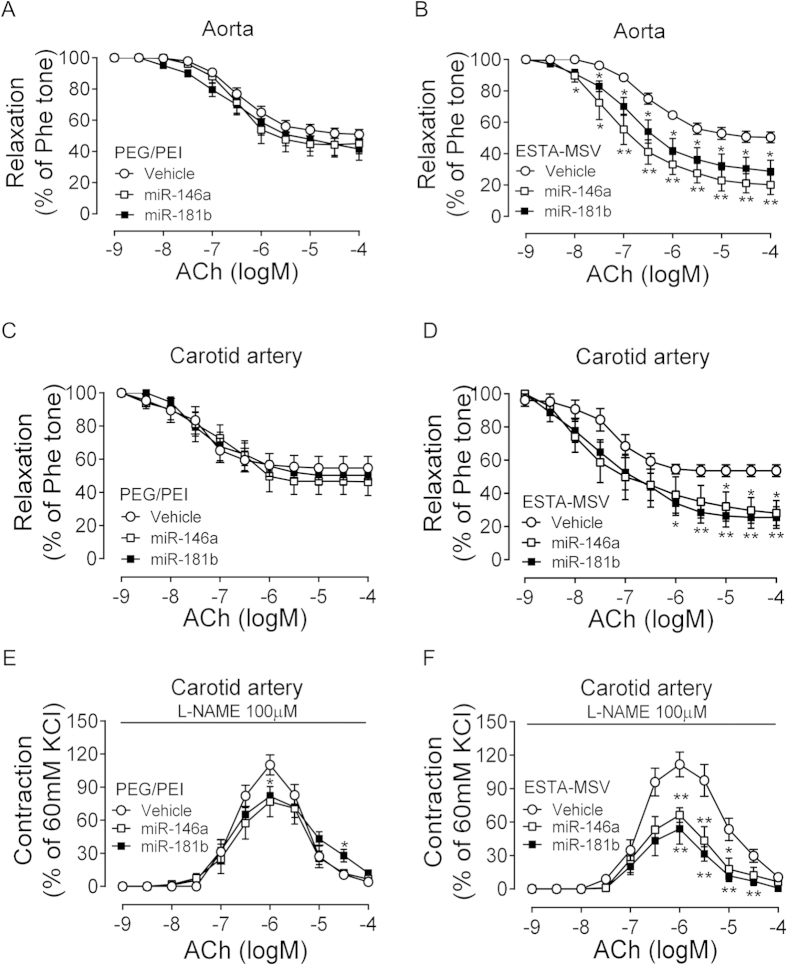
Effects of PEG/PEI/miRs and ESTA-MSV/miRs on the endothelial function of ApoE^−/−^ mice. The ACh-induced relaxation of Phe-precontracted abdominal aortas of ApoE^−/−^ mice after intravenously injected with vehicle, miR-146a, and miR-181b (15 μg) loaded in PEG/PEI nanoparticles (**A**) or ESTA-MSV microparticles (**B**) biweekly for 12 weeks. (**C**,**D**) The ACh-induced relaxation of carotid arteries of ApoE^−/−^ mice treated as above. (**E**,**F**) The ACh-induced contraction of carotid arteries of ApoE^−/−^ mice in the presence of L-NAME (100 μmol/L). Data are shown as the means ± SEM (n = 5). *P < 0.05, **P < 0.01 *vs.* vehicle group.

ApoE^−/−^ mice were fed a Western diet and intravenously injected with particles biweekly for 12 weeks. Considering the hypothesis that endothelial dysfunction is the initial atherogenic event, we began treatment during early atherogenesis, and assessed endothelial function by testing vascular reactivity in aortas and carotid arteries *ex vivo*. We found that ESTA-MSV/miRs, but not PEG/PEI/miRs, significantly improved the ACh-induced endothelium-dependent relaxation of both aortas and carotid arteries of ApoE^−/−^ mice (Fig. 3A–D). SNP-induced endothelium-independent relaxations in these arteries were comparable in all groups (Supplementary Fig. S4A–D). We also observed that the ESTA-MSV/miRs inhibited the ACh-induced contraction, a marker of endothelial dysfunction, in the presence of nitric oxide (NO) synthase inhibitor, while the PEG/PEI/miRs had little effect (Fig. 3E,F).

### ESTA-MSV/miRs decrease plaque size and stabilize plaques of ApoE^−/−^ mice

**Figure 4:**
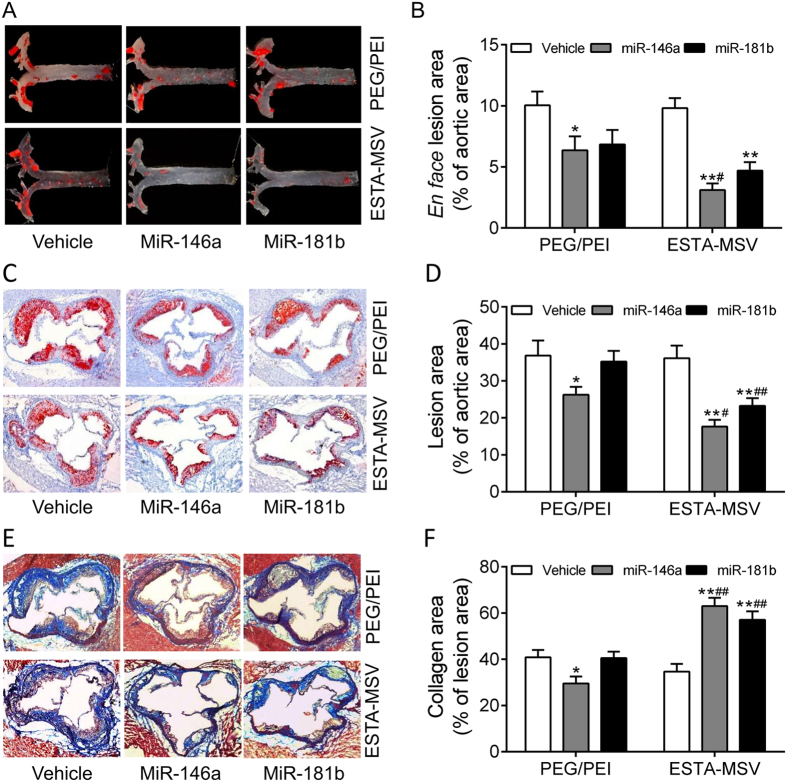
Effects of PEG/PEI/miRs and ESTA-MSV/miRs on the aortic atherosclerosis of ApoE^−/−^ mice. Representative *en face* ORO-stained aortic arches and thoracic aortas of ApoE^−/−^ mice intravenously injected with vehicle, miR-146a, and miR-181b (15 μg) loaded in PEG/PEI nanoparticles or ESTA-MSV microparticles (**A**) biweekly for 12 weeks. (**B**) Quantification of *en face* aortic plaque size. (**C**) Representative ORO-stained atherosclerotic lesions in frozen sections of aortic roots from ApoE^−/−^ mice. Magnification: 20X. (**D**) Quantification of plaque size in aortic root. (**E**) Representative Masson trichrome-stained collagen (blue) in aortic root. Magnification: 20X. (**F**) Quantification of collagen content. Data are shown as the means ± SEM (n = 5). *P < 0.05, **P < 0.01 *vs.* vehicle group of the same particle, ^#^*P* < 0.05, ^##^*P* < 0.01 *vs.* PEG/PEI groups of the miR.

In PEG/PEI/miRs-treated groups, plaque size was moderately lower compared to vehicle-treated group while according to the *en face* ORO-staining, both miRs packaged in ESTA-MSV microparticles significantly decreased plaque size in aortic arches and branches and in the thoracic aortas (Fig. 4A,B). However, the miR-181b packaged in MSV non-targeted system did not have effects, and so did scrambled RNA (Supplementary Fig. S5). ESTA-MSV-miR146a appeared to be the most potent effector of reduced lesion size. Similar results were obtained from the ORO-stained frozen sections of aortic roots (Fig. 4C,D). In addition, ESTA-MSV/miRs markedly increased the collagen deposition in plaques shown by Masson trichrome-stained sections of aortic roots (Fig. 4E,F).

### ESTA-MSV/miRs decrease macrophages in plaques of ApoE^−/−^ mice

**Figure 5:**
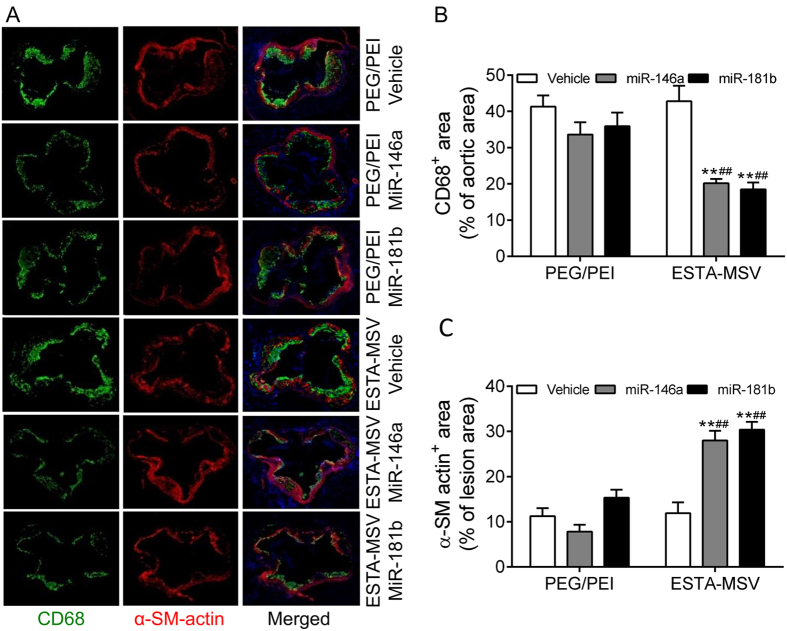
Effects of PEG/PEI/miRs and ESTA-MSV/miRs on the infiltration of macrophages and vascular SMCs in atherosclerotic lesions of ApoE^−/−^ mice. (**A**) Representative images of double immunofluorescence staining of CD68-positive macrophages (green) and α-SM-actin-positive vascular SMCs (red) in the aortic root atherosclerotic plaques of ApoE^−/−^ mice intravenously injected with vehicle, miR-146a, and miR-181b (15 μg) loaded in PEG/PEI nanoparticles or ESTA-MSV microparticles biweekly for 12 weeks. Magnification: 20X. The percentage of CD68-positive (**B**) and α-SM-actin-positive (**C**) area in the aortic root lesion. **P < 0.01 *vs.* vehicle group of the same particle, ^##^*P* < 0.01 *vs.* PEG/PEI groups of the miR.

According to double immunostaining plaques contained CD68-positive macrophages and α-SM-actin-positive vascular SMCs. What is more, the number of macrophages decreased while that of the vascular SMCs increased in plaques of ESTA-MSV/miRs-treated vs. vehicle-treated mice (Fig. 5A,B). However, PEG/PEI/miRs had no effect (Fig. 5A,B).

### ESTA-MSV/miRs downregulate adhesion molecules in aortas of ApoE^−/−^ mice

**Figure 6:**
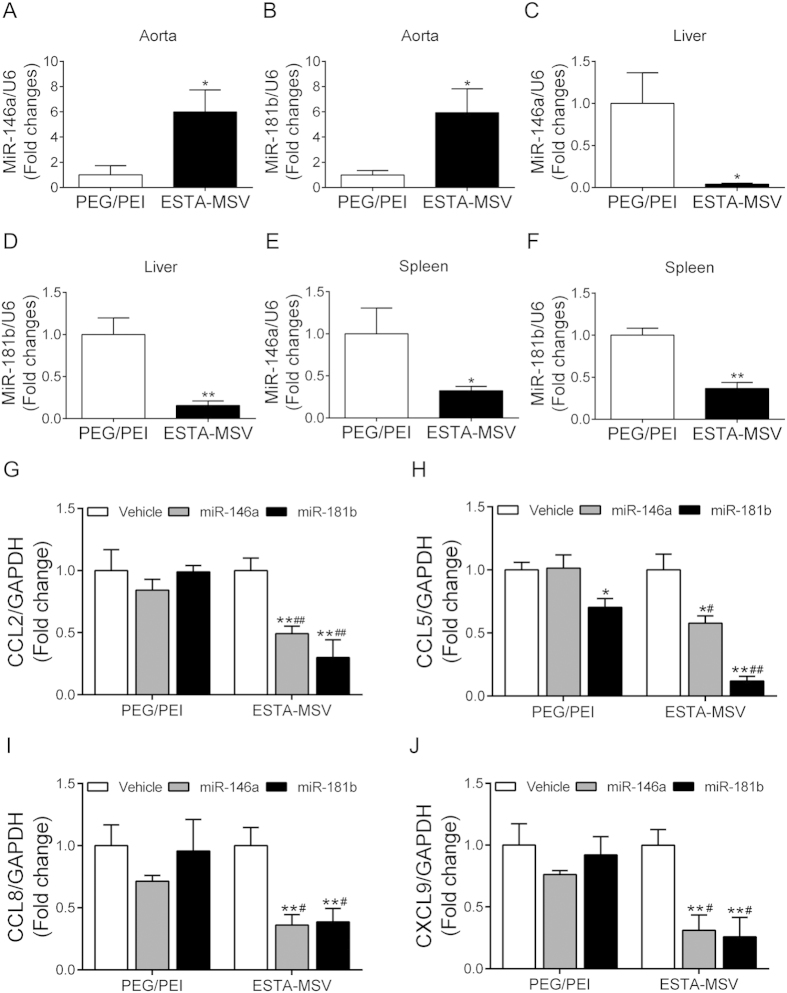
The expression of miR-146a/-181b and their target genes in aortic tissue of ApoE^−/−^ mice. The expression of miR-146a in aorta (**A**) liver (**C**) and spleen (**E**) of ApoE^−/−^ mice after intravenously injected with PEG/PEI/miR-146a or ESTA-MSV/miR-146a biweekly for 12 weeks. The expression of miR-181b in aorta (**B**) liver (**D**) and spleen (**F**) of ApoE^−/−^ mice after injected with PEG/PEI/miR-181b or ESTA-MSV/miR-181b for 12 weeks. *P < 0.05, **P < 0.01 *vs.* PEG/PEI group. The expression of Ccl2 (**G**), Ccl5 (**H**), Ccl8 (**I**), and Cxcl9 (**J**) in aortic tissues of ApoE^−/−^ mice after intravenously injected with vehicle, miR-146a, and miR-181b (15 μg) loaded in PEG/PEI nanoparticles or ESTA-MSV microparticles biweekly for 12 weeks. Data are shown as the means ± SEM (n = 5). *P < 0.05, **P < 0.01 *vs.* vehicle group of the same particle, ^#^*P* < 0.05, ^##^*P* < 0.01 *vs.* PEG/PEI groups of the miR.

Aortic tissue-miR-146a/-181b expression was higher in ESTA-MSV- vs. PEG/PEI-treated mice, whereas the opposite occurred in the liver and spleen, which are the major sites of PEG/PEI-miRNAs deposition (Fig. 6A–F). In addition, expression of Ccl2, Ccl5, Ccl8, and Cxcl9 in the abdominal aortas was lower in both ESTA-MSV/miRs-treated vs. vehicle-treated control mice (Fig. 6G–J), while PEG/PEI/miRs only minimally affected expression of these chemokines (Fig. 6G–J). Neither microparticle treatment affected the serum lipids nor the glucose tolerance in mice (Supplementary Fig. S6A–D) nor the serum ALT and AST activities (Supplementary Fig. S7A,B) and serum creatinine level (Supplementary Fig. S7C).

## Discussion

We described a novel and effective targeted delivery system for miRs-based therapy for atherosclerosis. Although the miRs have potential in the treatment of atherosclerotic diseases^19^, critical challenges about *in vivo* delivery must be overcome prior to its translation to clinical applications; e.g., once entering the blood stream, miRs can be rapidly cleared. Several viral vectors and non-viral delivery systems have been formulated to address the limitations of poor *in vivo* stability and inappropriate biodistribution^20^. Compared with viral vectors and liposomes, the PEG/PEI nanoparticles are a safer and more efficacious delivery system *in vivo*^11^,^21^,^22^, although non-specific distribution has been an unresolved problem. The ESTA-MSV targeting delivery system was previously used to treat bone marrow metastatic breast cancer^15^. However, this is the first attempt to inhibit endothelial inflammation and atherosclerosis via an E-selectin-targeting carrier to deliver miRs. In this study, the ESTA-MSV microparticle-packaged miRs showed excellent therapeutic effects on atherosclerotic lesions; while non-specific PEG/PEI nanoparticle-packaged miRs had little or no effect, a difference that may be attributed to the differential distribution due to ESTA-MSV affinity. Compared to PEG/PEI, the ESTA-MSV-mediated delivery gave higher, stable expression of exogenous miRs in aorta but lower levels in liver and spleen thereby indicating specificity even if delivered systemically. Our novel findings suggest that the ESTA-MSV delivery system not only has higher selectivity of distribution but also carries the agents for controlled release.

Sun *et al*.^23^ reported that injection with 1 nmol of miR-181b packaged with lipofectamine once a week for 12 weeks through tail vein attenuated the atherosclerosis in ApoE^−/−^ mice. In the present study, mice were injected with 2 nmol of miR-181b packaged in ESTA-MSV system every other week and showed decreased atherosclerosis. The average dosage of miR-181b was similar between these two studies. It suggests that ESTA-MSV may achieve pharmacological effects with a lower administration frequency.

E-selectin, also known as endothelial-leukocyte adhesion molecule 1, is a cell adhesion molecule expressed on inflamed endothelial cells in response to inflammatory cytokines^24^,^25^. We confirmed, in this study, that the E-selectin is almost absent in healthy endothelium but is apparently induced in irregular endothelium covering atherosclerotic plaques in mice and humans. E-selectin is also expressed in newly-formed microvessels, which are usually within an inflammatory microenvironment. This feature makes it feasible in treating bone marrow metastatic breast cancer by targeting the tumor neovessels as in our previous study^15^. Neovascularization also exists in the established atherosclerotic plaques, and is thought to promote lesion progression, including destabilization, hemorrhage, erosion and ultimately rupture^26^,^27^. Therefore, inhibition or normalization of the plaque angiogenesis to stabilize the lesion could be the mechanism underlying the therapeutic effectiveness of ESTA-MSV^28^. However, miRs delivery to neovessels within the plaque via ESTA-MSV microparticles remains an open question worth addressing. This hypothesis requires further investigation of the ESTA-MSV delivery system in established atherosclerotic plaques.

Several kinds of nanoparticles and microparticles have been developed and tested in the experimental atherosclerosis. Based on their physical features, PLGA-b-PEG copolymer and sugar-based amphiphilic nanoparticles should target macrophages^29^,^30^. PLGA-b-PEG copolymer encapsulated liver X receptor agonist delivery in LDL receptor^−/−^ mice shows localized effects on macrophage without targeting liver thus sparing adverse effect on lipid metabolism^29^. A sugar-based amphililic macromolecule effectively blocks oxidized LDL uptake through binding and regulating macrophage scavenger receptors^30^. A nanoparticle carrier system (MaxSuppressor *In vivo* RNA Lancer Kit) delivers miR-126-5p to vascular ECs and reduces atherosclerosis^31^. Moreover, many attempts have been made to modify the particle surface—ICAM1-targeting, VCAM1-targeting, collagen IV-targeting, and vascular targeting particles—so that they target the plaque^32^,^33^,^34^,^35^,^36^. VCAM-1-targeted particles carried and delivered miR inhibitors to ECs and prevented atheroma formation in a mouse model of atherosclerosis^37^,^38^. Collagen IV-targeted nanoparticles delivered a small peptide, which would otherwise have been quickly renally cleared, to advanced plaques^34^. Another peptide, PREY, which has high fibronectin and filamin-A affinity when conjugated to liposomes, targets the atheroprone vasculature by delivering BH_4_, which reduces vascular superoxide^35^. Additionally, silica-gold nanoparticle alone may achieve therapeutic effects without anti-atherosclerotic agents in patients diagnosed with coronary artery disease^39^. The present study identified the ESTA-MSV microparticle as promising therapeutic alternative to combat atherosclerosis.

MiRs are endogenous small non-coding RNAs (containing ~22 nucleotides), which function in post-transcriptional regulation of gene expression^40^. Several miRs have been identified as regulators of EC, vascular SMC, and macrophage functions, and thereby regulate the development and progression of atherosclerosis^41^. Moreover, the miRs have emerged as potent therapeutic agents for atherosclerosis. In endothelial cells, miR-146a and miR-181b produced anti-inflammatory effects by suppressing the nuclear factor-κB pathway^23^,^42^,^43^. In the present study, we found that the atheroprotective effects of these two shear stress-inducible miRs are mediated through downregulated adhesion molecule expression, which reduces adhesion of monocytes to ECs. However, we could not exclude other possible mechanisms. As these two miRs may also be involved in the glucose and lipid metabolism^44^, we evaluated the serum lipid levels and glucose tolerance and detect no differences among the groups.

Impairment ACh-induced relaxation and enhanced ACh-induced contraction are markers of endothelial dysfunction. Although the mechanisms underlying the miRs-elicited improvement of endothelial function in ApoE^−/−^ mice are not thoroughly understood, one might assume that the inhibition of macrophage infiltration and inflammation of vascular wall might improve NO-dependent vasodilation. In addition, miR-146a also targets NADPH oxidases that produce reactive oxygen species (ROS)^45^,^46^, a major NO quencher that is important role in the regulation of endothelial function. Therefore, ROS inhibition might improve endothelial NO-mediated vasodilation and contribute to the atheroprotective effect of miR-146a; this needs further investigation.

Although the ESTA-MSV system was designed to specifically target E-selectin on inflamed endothelium, we cannot rule out the participation of other types of cells, which may also be affected, including SMCs and macrophages in the vascular wall.

In summary, given that E-selectin expression is enhanced on inflamed EC surface, E-selectin-targeting ESTA-MSV microparticles enrich endothelium with therapeutic miRs. Importantly, we found that miR-146a and miR-181b decreased plaque size and macrophage infiltration, while increased vascular SMCs and collagen deposition.

## Additional Information

**How to cite this article**: Ma, S. *et al*. E-selectin-targeting delivery of microRNAs by microparticles ameliorates endothelial inflammation and atherosclerosis. *Sci. Rep.* **6**, 22910; doi: 10.1038/srep22910 (2016).

## Supplementary Material

Supplementary Information
