# Supplementary material for: E-selectin-targeting delivery of microRNAs by microparticles ameliorates endothelial inflammation and atherosclerosis
Source: Sci Rep. 2016 Mar 9;6:22910. doi: 10.1038/srep22910 (PMC4783714; doi:10.1038/srep22910)
Supplement: Supplementary Information [file srep22910-s1.doc]

**Supplemental information**

**E-selectin-targeting delivery of microRNAs by microparticles ameliorates endothelial inflammation and atherosclerosis**

Shuangtao Ma*^,1^, Xiao Yu Tian*^,1,2^, Yunrong Zhang^1^, Chaofeng Mu^3^, Haifa Shen^3^, Jean Bismuth^4^, Henry J. Pownall^1^, Yu Huang^2^ & Wing Tak Wong^1^

^1^Department of Cardiovascular Sciences, Houston Methodist Research Institute, Houston, TX 77030, USA. ^2^School of Biomedical Sciences and Li Ka Shing Institute of Health Sciences, Chinese University of Hong Kong, Shatin, NT, Hong Kong SAR, China. ^3^Department of Nanomedicine, Houston Methodist Research Institute, Houston, TX 77030, USA. ^4^Methodist DeBakey Heart & Vascular Center, Houston Methodist Hospital, Houston, TX 77030, USA.

*These authors contributed equally to this work.

Correspondence and requests for materials should be addressed to W.T.W. (email: wwong@houstonmethodist.org).

**Supplemental Table 1.** Primers for real-time qPCR

| Name | Sequence (5’-3’) |
| --- | --- |
| Human E-selectin forward | TGGCTTCAGTGGACTCAAGT |
| Human E-selectin reverse | GGTAACCCCTATCACAGCTGA |
| Human VCAM1 forward | AAGATGGTCGTGATCCTTGG |
| Human VCAM1 reverse | GGTGCTGCAAGTCAATGAGA |
| Human ICAM1 forward | CAGAGGTTGAACCCCACAGT |
| Human ICAM1 reverse | TCCTCTGGCTTCGTCAGAATC |
| Human CCL2 forward | CAGCCAGATGCAATCAATGCC |
| Human CCL2 reverse | TGGAATCCTGAACCCACTTCT |
| Human CCL5 forward | CCCAGCAGTCGTCTTTGTCA |
| Human CCL5 reverse | TCCCGAACCCATTTCTTCTCT |
| Human CCL8 forward | TGGAGAGCTACACAAGAATCACC |
| Human CCL8 reverse | TGGTCCAGATGCTTCATGGAA |
| Human CXCL9 forward | CCAAGGGACTATCCACCTACAATC |
| Human CXCL9 reverse | GGTTTAGACATGTTTGAACTCCATTC |
| Human GAPDH forward | GTGAAGGTCGGAGTCAACG |
| Human GAPDH reverse | TGAGGTCAATGAAGGGGTC |
| Mouse Ccl2 forward | CATCCACGTGTTGGCTCA |
| Mouse Ccl2 reverse | GATCATCTTGCTGGTGAATGAGT |
| Mouse Ccl5 forward | AGATVTCTGCAGCTGCCCTCA |
| Mouse Ccl5 reverse | GGAGCACTTGCTGCTGGTGTAG |
| Mouse Ccl8 forward | TTCTTTGCCTGCTGCTCATA |
| Mouse Ccl8 reverse | AGCAGGTGACTGGAGCCTTA |
| Mouse Cxcl9 forward | TCC TTT TGG GCA TCA TCT TC |
| Mouse Cxcl9 reverse | TTC CCC CTC TTT TGC TTT TT |
| Mouse Gapdh forward | AGGTCGGTGTGAACGGATTTG |
| Mouse Gapdh reverse | TGTAGACCATGTAGTTGAGGTCA |

VCAM1, vascular cell adhesion molecule 1; ICAM1, intercellular adhesion molecule 1.

**A**

**B**

**Supplementary Fig. 1.** The expression of miR-146a/-181b in human aortas. The expression of miR-146a (A) and miR-181b (B) in aortic tissues with atherosclerotic plaque or without plaque from patients with aortic atherosclerotic aneurysm. *P<0.05 vs. aortas without plaques. n=4 in each group.

**A**

**B**

**Supplementary Fig. 2.** The expression of adhesion molecules in inflamed endothelial cells. The expression of vascular cell adhesion molecule 1 (VCAM1, A) and intercellular adhesion molecule 1 (ICAM1, B) in human microvascular endothelial cells after treatment with tumor necrosis factor (TNF)-α (10ng/mL) for 0, 2, and 6 hours. **P<0.01 vs. 0h.


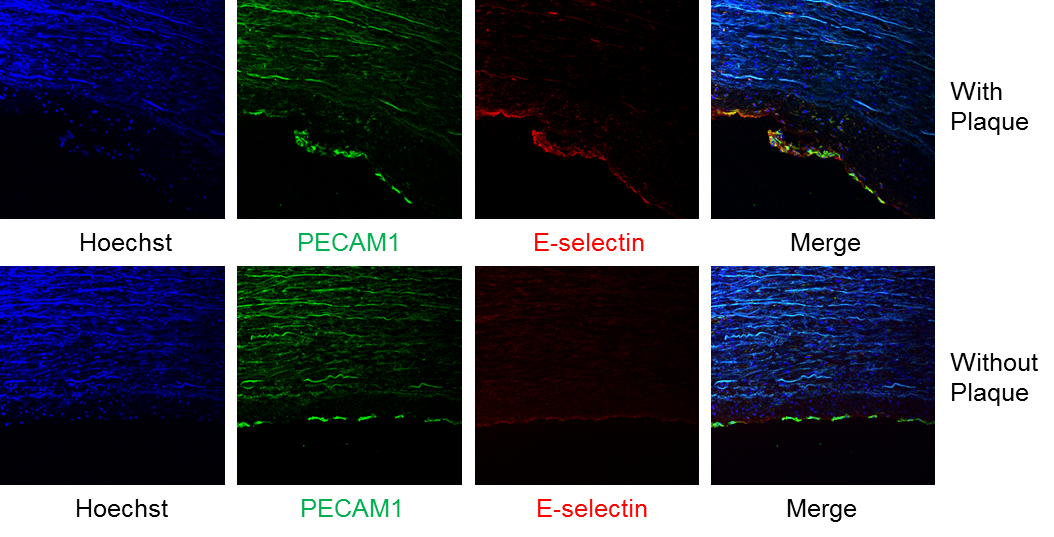


**Supplementary Fig. 3.** The expression of E-selectin in human aorta. Representative images of double immunofluorescence staining of endothelial cell marker platelet endothelial cell adhesion molecule 1 (PECAM1, green) and E-selectin (red) in human aortas with plaque (upper panel) and without plaque (lower panel).

A B

C D

**Supplementary Fig. 4.** The sodium nitroprusside (SNP)-induced vasodilation. The SNP-induced relaxation of aortas of apolipoprotein E-deficient (ApoE^-/-^) mice after intravenously injected with vehicle, miR-146a, and miR-181b (15µg) loaded in PEG/PEI nanoparticles (A) or ESTA-MSV microparticles (B) biweekly for 12 weeks. C and D, The SNP-induced relaxation of carotid arteries of ApoE^-/-^ mice treated as above.


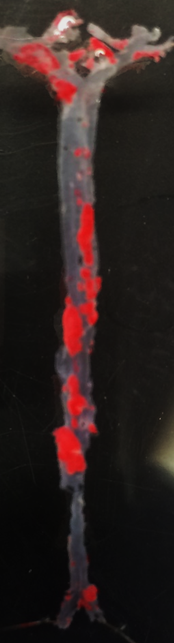

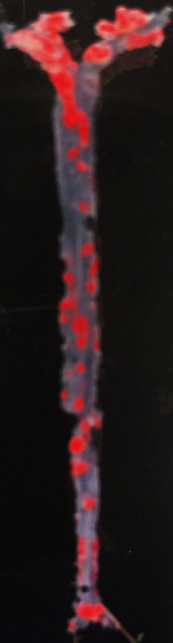

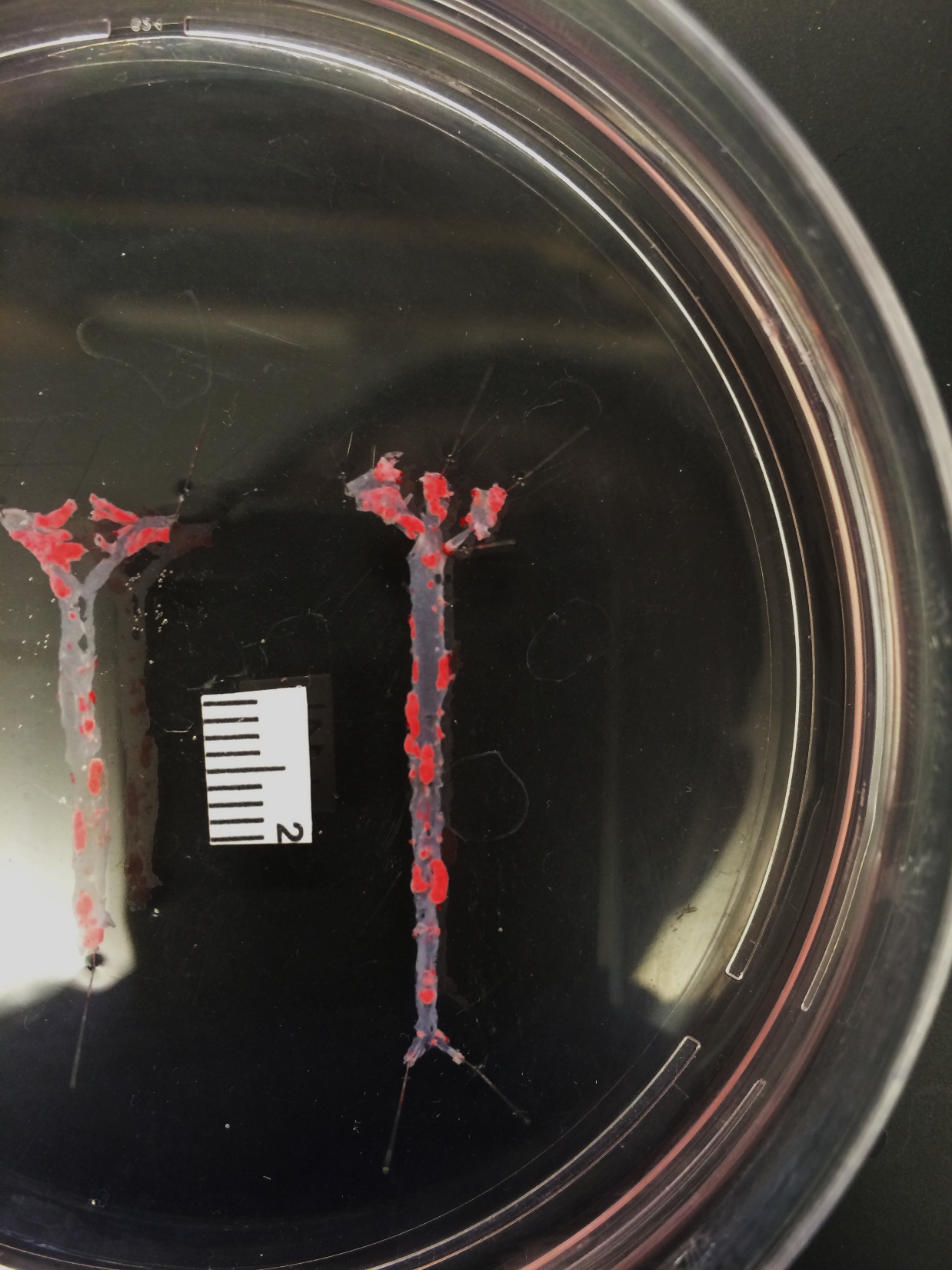


MSV-Vehicle

MSV-miR-181b

MSV-scra-RNA

**Supplementary Fig. 5.** *En face* oil red O-staining of mouse aortas. The oil red O-stained en face aortic preparations of apolipoprotein E-deficient (ApoE^-/-^) mice after intravenously injected with vehicle, scrambled (scra) RNA (15µg), and miR-181b (15µg) loaded in MSV non-specific microparticles biweekly for 12 weeks. n=3 per group.

A B

C D

**Supplementary Fig. 6.** The glycolipid profiles of apolipoprotein E-deficient (ApoE^-/-^) mice. The triglyceride (TG), total cholesterol (TC), low-density lipoprotein cholesterol (LDL-C), and high-density cholesterol (HDL-C) of ApoE^-/-^ mice after intravenously injected with vehicle, miR-146a, and miR-181b (15µg) loaded in PEG/PEI nanoparticles (A) or ESTA-MSV microparticles (B) biweekly for 12 weeks. C and D, The intraperitoneal glucose tolerance tests of ApoE^-/-^ mice treated as above.

A

B

C

**Supplementary Fig. 7.** Hepatic and renal function of apolipoprotein E-deficient (ApoE^-/-^) mice. The serum alanine transaminase (ALT, A), aspartate aminotransferase (AST, B), and creatinine (C) levels of ApoE^-/-^ mice after intravenously injected with vehicle, miR-146a, and miR-181b (15µg) loaded in PEG/PEI nanoparticles or ESTA-MSV microparticles biweekly for 12 weeks.
